# Studies on the Genetic Variation of the Green Unicellular Alga *Haematococcus pluvialis* (Chlorophyceae) Obtained from Different Geographical Locations Using ISSR and RAPD Molecular Marker 

**DOI:** 10.3390/molecules16032599

**Published:** 2011-03-22

**Authors:** Noroozi Mostafa, Hishamuddin Omar, Soon Guan Tan, Suhaimi Napis

**Affiliations:** 1 Department of Biology, Faculty of Science, University Putra Malaysia, 43400 Serdang, Selangor, Malaysia; 2 Department of Biology Faculty of Science, Alzahra University, Tehran 1993891176, Iran; 3 Department of Cell and Molecular Biology, Faculty of Biotechnology and Biomolecular Science, University Putra Malaysia, 43400 Serdang, Selangor, Malaysia

**Keywords:** genetic diversity, *Haematococcus*, ISSR, RAPD, strains, geographical

## Abstract

*Haematococcus pluvialis* (Flotow) is a unicellular green alga, which is considered to be the best astaxanthin-producing organism. Molecular markers are suitable tools for the purpose of finding out genetic variations in organisms; however there have been no studies conducted on ISSR or RAPD molecular markers for this organism. The DNA of 10 different strains of *H. pluvialis* (four strains from Iran, two strains from Finland, one strain from Switzerland and three strains from the USA) was extracted. A genetic similarity study was carried out using 14 ISSR and 12 RAPD primers. Moreover, the molecular weights of the bands produced ranged from 0.14 to 3.4 Kb. The PCA and dendrogram clustered the *H. pluvialis* strains into various groups according to their geographical origin. The lowest genetic similarity was between the Iran2 and USA2 strains (0.08) and the highest genetic similarity was between Finland1 and Finland2 (0.64). The maximum numbers of bands produced by the ISSR and RAPD primers were 35 and 6 bands, respectively. The results showed that ISSR and RAPD markers are useful for genetic diversity studies of *Haematococcus* as they showed geographical discrimination.

## 1. Introduction

Algae are one of the most useful natural resources that can be used to produce different bioactive compounds such as vitamins, proteins, unsaturated fatty acids, antioxidants and carotenoids, including astaxanthin. During the past two decades, scientists have discovered that *Haematococcus*, a unicellular green alga, is the best source of organisms that produce astaxanthin, the most powerful naturally occurring antioxidant. Astaxanthin can be used as a preventive medicine, by being able to slow down degenerative diseases and cardiovascular problems, having anti-cancer and anti-immunological disease properties and finally, its ability to stimulate the proliferation of neural progenitor cells to recover stem cell function [1,2,3,4]. 

Morphological traits observed through the light microscope have been traditionally used to determine the species and the diversity of *Haematococcus*, which has a complex life cycle with different morphological stages affected by environmental conditions. The morphology alone is not able to recognize strains which have various shapes in diverse environmental conditions and the cryptic species (due to recent speciation) with similar morphological traits however they are different genetically [5]. Molecular and genetic characters are affected less than the morphological characters by environmental conditions, hence they are more stable [6]. In addition to the necessity of the morphological study, there is a need to the molecular study of organisms in order to differentiate them geographically [7]. The combination of molecular and morphology provide a robust way to determine organisms with lower mistakes.

Biotechnological methods and molecular markers are great promising tools for improvement and enhancement of biomass production, astaxanthin production and tolerance to stresses in *H. pluvialis*. Most of the molecular marker tools are valuable methods to investigate population genetic and diversity which were developed quickly over the three past decades [8]. There have been some studies on algae using Inter Simple Sequence Repeat (ISSR) and Random Amplified Polymorphic DNA (RAPD) molecular markers [9,10,11,12], however, there have been none thus far on *H. pluvialis.* Therefore, this study was conducted for the first time with the aim of remedying this situation. ISSR markers are reliable, highly polymorphic, low cost and less laborious, need only a small amount of DNA and are very fast when compared to most other molecular markers [13]. ISSR and RAPD do not require DNA sequence data and in terms of reproducibility, ISSR is comparable to SSR [14]. The RAPD technique has wide applications in breeding, genetic evolution, gene mapping and population genetics and is able to produce many markers with low cost and high speed. Although the reproducibility of RAPD technique is low and is dominant, it is one of the important molecular markers [15]. The ISSR technique is a dominant marker too but its reproducibility is higher than RAPD. Molecular and genetic study of any organism needs pure and axenic cultures whereas the growth of *Haematococcus* is very difficult due to their sensitivity to contamination. The pH of medium is neutral and other algal species, bacteria or fungi easily can dominate and make a culture fail. The molecular markers are able to distinguish other strains of *H. pluvialis* with desirable properties from various parts of the world.

The objective of this study was to find out the genetic diversity of the green unicellular alga, *Haematococcus pluvialis* by using ISSR and RAPD markers. There is a tendency to depend on the culture collection institutes that represents a limitation for scientists. This dependency on culture collections can deprive researchers from access to new species and strains with diverse characteristics and various bioactive compounds which can be found in other habitats. Four new strains were isolated from different cities of Iran in order to examine their diversity and to uncover their differences with CCAP (Culture Collection of Algae and Protozoa) strains, using molecular markers. If useful the new strains isolated from Iran water bodies could be deposited in culture collections in order to enrich the gene reserves.

## 2. Results and Discussion

**Figure 1:**
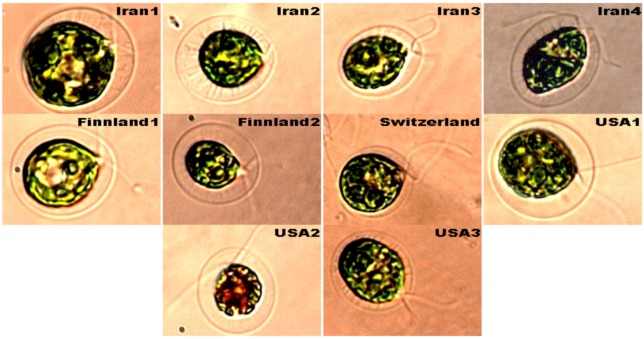
The morphology of ten strains of *H. pluvialis.*

Four strains of *H. pluvialis* (named Iran1, 2, 3, 4) were isolated from Iran and six other strains (Finland1, 2, Switzerland and USA1, 2, 3) were purchased from CCAP. The images of ten strains of *H. pluvialis* are depicted in Figure 1, which shows their similarity, despite small differences that are related to the complex life cycle of *H. pluvialis* which has four different morphological stages. 

**Figure 2:**
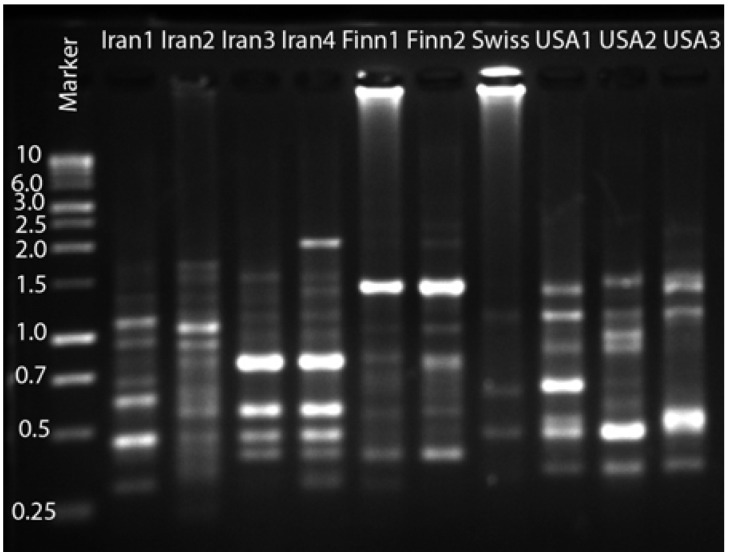
The bands produced by ISSR primer14 for 10 Strains of *H. pluvialis.*

The amplification of *Haematococcus* DNA using 14 ISSR primers produced 317 scoreable bands and using 12 RAPD primers, 115 bands were produced. Figure 2 shows the agarose gel electrophoresis and polymorphic bands produced by ISSR primer 14 for a number of 10 strains of *H. pluvialis*. The similarity and differences between strains are visible in this figure.

In Table 1, the Jacard’s similarity index among 10 strains of *H. pluvialis* is presented. As can be seen from the table, the minimum amount of Jacard’s similarity was 0.08 between Iran2 and USA2 which were from two distinct geographical areas and the maximum 0.64 was between Finland1 and Finland2 strains. The same conclusion obtained from Nei’s genetic identity, which was 0.86 between Iran3 and Iran4 and 0.9 between Finland1 and Finland2. The polymorphism level for pooled data of ISSR an RAPD markers was 98.6%. 

**Table 1:** The Jacard’s similarity index among ten strains of *Haematococcus pluvialis.*

Strains	Jacard’s similarity Index									
	Iran1	Iran2	Iran3	Iran4	Finland1	Finland2	Switzer-land	USA1	USA2	USA3
**Iran1**	1.00									
**Iran2**	0.47	1.00								
**Iran3**	0.23	0.24	1.00							
**Iran4**	0.23	0.22	0.57	1.00						
**Finland1**	0.11	0.11	0.14	0.19	1.00					
**Finland2**	0.10	0.11	0.14	0.16	0.64^*^	1.00				
**Switzerland **	0.13	0.10	0.14	0.14	0.16	0.17	1.00			
**USA1**	0.14	0.08^*^	0.16	0.12	0.14	0.16	0.32	1.00		
**USA2**	0.16	0.13	0.15	0.12	0.14	0.17	0.25	0.35	1.00	
**USA3**	0.20	0.14	0.19	0.14	0.16	0.16	0.22	0.36	0.35	1.00

The results presented in Table 2 indicate the polymorphisms among the studied *Haematococcus pluvialis* strains. It should be noted that the molecular weights of the bands ranged from 0.14 to 3.4 Kb. This table depicts the total number of bands produced, their polymorphism, sequence and annealing temperature for 26 primers. Most of the primers produced bands that were 100% polymorphic and their annealing temperature varied from 45 °C to 67 °C. Maximum bands were obtained by ISSR primers (35 and 29 in primers 5 and 4) and the average number of band produced by the ISSR and RAPD primers were 23 and 10 respectively which means that ISSR primers produced more polymorphic bands than RAPD primers and it can be more powerful tool to investigate biodiversity. The higher annealing temperatures will provide the higher reproducibility and specific attachment of the primers to the template. Table 2 depicted that the maximum annealing temperature of ISSR and RAPD primer was 67 °C and 54 °C respectively.

The value of the average Shannon’s indices for ISSR was 0.509 with a standard deviation of 0.144. The AMOVA test for the bands generated by the ISSR and RAPD primers were 74% within the populations and 26% among the populations (p < 0.01). On the other word based on the common loci investigated only 26% of the total variation in the *Haematococcus pluvialis* may be attributed to variation among the strains.

**Table 2:** Summary of characteristics of primers and ratio of polymorphic ISSR and RAPD loci and Fragment size range (Kb) in *H.pluvialis.*

	Primer Name	Primer Sequence	Scored Bands	Ratio of Polymorphic Bands	Fragment Size range (Kb)	Annealing Temperature
1	ISSR 1	(CCA)_5_	25	24/25(96%)	0.26–2.53	60
2	ISSR 4	(ATG)_5_	13	13/13(100%)	0.45–1.19	50
3	ISSR 5	GTC(CT)_8_	19	19/19(100%)	0.46–2.79	60
4	ISSR 7	GGGC(GA)_8_	29	29/29(100%)	0.29–2.57	67
5	ISSR 10	(CAC)_4_RC	35	35/35(100%)*	0.44–2.68	57
6	ISSR 11	(GTG)_3_GC	26	26/26(100%)	0.36–2.54	60
7	ISSR 12	(GAG)_3_GC	22	22/22(100%)	0.47–2.1	58
8	ISSR 13	(GT)_6_GG	15	15/15(100%)	0.48–2.06	55
9	ISSR 14	(GA)_6_CC	24	22/24(91.6%)	0.14–2.11	55
10	ISSR 15	(GT)_6_CC	16	16/16(100%)	0.24–0.92	59
11	ISSR 16	(AC)_8_C	21	21/21(100%)	0.49–3.42	59
12	ISSR 17	(GA)_8_C	24	24/24(100%)	0.33–2.05	59
13	ISSR 18	(GA)_8_T	26	26/26(100%)	0.14–.052	57
14	ISSR 19	(GACA)_4_	22	22/22(100%)	0.15–2.59	58
15	RAPD 1	ACA.ACT.GCT.C	7	7/7(100%)	0.42–1.44	37
16	RAPD 2	TGA.CTG.ACG.C	6	5/6(83%)	o.35–2.1	42
17	RAPD 3	GCG.ATC.CCC.A	7	9/9(100%)	0.48–1.7	44
18	RAPD 4	ATC.GGG.TCC.G	8	9/10(90%)	0.62–2.16	45
19	RAPD 5	CAG.GCC.CTT.C	11	17/17(100%)	0.22–1.86	43
20	RAPD 6	AAT.CGG.GCT.G	20	20/20 (100%)*	0.43–1.8	42
21	RAPD 7	GGG.TAA.CGC.C	10	6/6(100%)	0.72–2.4	38
22	RAPD 8	CAA.TCG.CCG.T	7	13/13(100%)	0.59–2.28	42
23	RAPD 9	CAG.CAC.CCA.C	6	9/9(100%)	0.62–1.51	54
24	RAPD 10	AGG.TGA.CCG.T	17	7/7(100%)	0.17–1.2	40
25	RAPD 11	AGC.GCC.ATT.G	17	11/11(100%)	0.22–0.92	42
26	RAPD 12	GAG.AGC.CAA.C	11	19/19(100%)	0.51–2.1	32

* The highest polymorphic ratio in ISSR and RAPD

**Figure 3:**
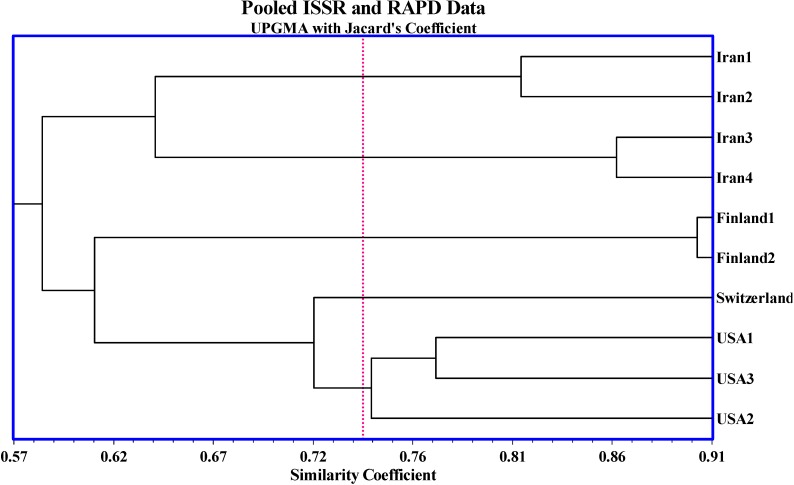
UPGMA Dendrogram of 10 strains of *H. pluvialis* based on mixed data of ISSR and RAPD markers.

**Figure 4:**
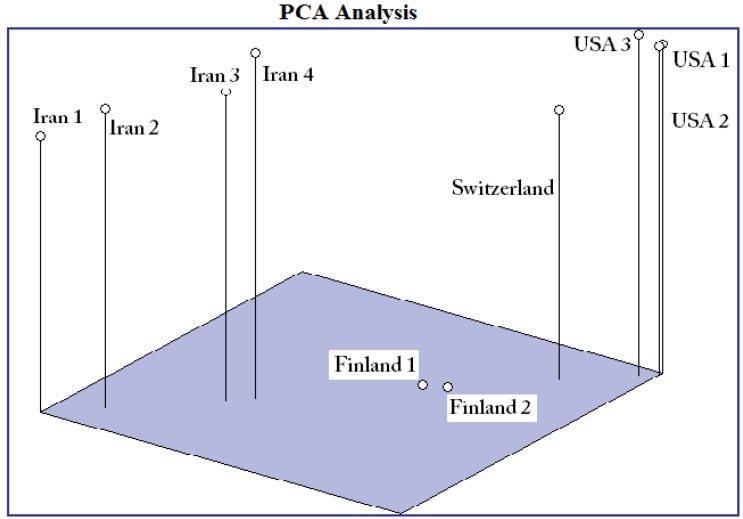
PCA analysis for 10 strains of *H. pluvialis.*

The algal strains belonging to the different geographical populations clustered separately according to the dendrogram in Figure 3 and PCA analysis Figure 4. The dendrogram constructed using the UPGMA method based on Jacard’s similarity matrix showed the genetic variation among the ten *H. pluvialis* strains studied. The *Alexandrium minutum* species showed high genetic diversity using the RAPD technique, even between strains from same bloom [16]. ISSR-PCR is a quick, produce sufficient polymorphism and reliable fingerprint method to distinguish potato cultivars [17]. The regression and association between the genetic diversity and geographical locations were visible in many cases [18].

The dendrogram distinctly separated the algae into five clusters based on the 0.74 reference line, with the strains from Iran making up two clusters, Finland one cluster, Switzerland one cluster and USA another cluster, therefore the dendrogram successfully grouped all the *H. pluvialis* strains based on their geographical origins (Figure 3). The results of the UPGMA and Neighbor Join dendrograms whether based on the ISSR and RAPD marker data separately (data not shown) or the combined (pooled) ISSR-RAPD markers were generally similar and supported one another with minor differences. For example, the ISSR lonely and pooled ISSR-RAPD dendrograms distinguished clearly between the ten strains however in the RAPD data based dendrogram, the Finland1, 2 were clustered with the Iran major group. This small difference could be due to the different primer attachment sites of the two marker systems in the algal genome. 

Figure 4 shows the PCA analysis where strains of *H. pluvialis* from different countries were grouped in four separate places and confirm the results of dendrogram. The Switzerland strain was grouped in the major branch of USA and it is more similar to the USA strain. 

The diversity in different strains of *H. pluvialis* was high, based on both ISSR and RAPD molecular markers. Bhau [19] reported that RAPD markers were more informative than ISSR markers whilst others have reported that ISSR is more efficient than RAPD [13,20,22].

According to this study, ISSR markers for *Haematococcus pluvialis* strains were more diverse as they produced more polymorphic bands with higher annealing temperature however its results were the same as the RAPD results confirming each other with small differences. It is important to find different strains of *H. pluvialis* to discover the source of useful genes and desired characteristics such as a thin cell wall or high growth rate and high astaxanthin production.

## 3. Experimental

### 3.1. General

The strains of *Haematococcus pluvialis* were gathered from the three continents of Asia, Europe and America. Four strains (Iran1, 2, 3, 4) were isolated from freshwater ponds located in the cities of Tehran and Karaj in Iran (Table 3) and six strains which originated from fresh water bodies in Finland, Switzerland and USA were obtained from the CCAP in the United Kingdom. The Finland strains were isolated from fresh water bodies in the Ostpicken Island (Tvärminne) and the USA strains were isolated from fresh water bodies in the states of Kansas, Virginia and Maryland and the Swiss strain was collected from the botanical garden of the University of Basel. 

**Table 3:** The geographic location of Iranian sampling stations where *H. pluvialis* were found.

**Station**	Golshahr Park (Karaj)	Kuye Karmandane shomali (Karaj)	Kuye Modares (Karaj)	Shemiranat (Tehran)
**E (Longitude)**	50˚59′13″	50˚58′44″	50˚59′15″	51˚23′51″
**N (Latitude)**	35˚49′17″	35˚49′ 56″	35˚50′33″	35˚47′45″

The algae were cultured in axenic and photoautotrophic condition at 23 °C and 40 μmol photons m^−2^ s^−1^ in light dark circadian cycle: 12-12 hours in aerating liquid Bold medium to get enough biomass for DNA extraction.

### 3.2. DNA Isolation and PCR

Genomic DNA was extracted from *H. pluvialis* using the CTAB method [23] and DNA quality and concentration were detected using a nanophotometer (Impelen Company) and was confirmed with 1% agarose gel electrophoresis. ISSR and RAPD primer sequences were acquired from different reference papers [9,10,11,21]. In total, 14 of 20 anchored and non-anchored ISSR primers and 12 of 18 RAPD primers generated DNA amplification products. Polymerase chain reaction (PCR) was performed using 20 µL of reaction mixture solution containing 11.5 µL of autoclaved distilled water, 2 µL of 10X PCR buffer, 1.4 µL of MgCl_2_ (50 mM), 0.6 µL of primer (5 mM), 0.4 µL of dNTP (25 mM), 1.6 µL of genomic DNA, 0.5 U of Taq DNA polymerase (Invitrogen) and 2 µL of Q-solution. PCR program used was: 97 °C for 6 min followed by 35 cycles of 94 °C for 45s, 48 °C to 69 °C for 45 s, 72 °C for 60 s and 72 °C for 5 min. The PCR protocol was same for ISSR and RAPD except annealing temperature of primers which were different. 

### 3.3. Data Analysis

Each of the resulting ISSR and RAPD bands were scored using the UVIDoc version 99.02 software and the data matrix constructed with bands indicated by (0) for absence and (1) for presence. The genetic similarity, dendrogram and the PCA analysis were obtained by importing the data matrix from Excel to NTSYS pc version 2.10e [25]. Jacard’s similarity index is suitable for dominant markers to detect strain similarities [26]. NJoin and SAHN (sequential agglomerative hierarchical nested clustering) in clustering option were employed to generate the dendrogram to illustrate the relationships among the strains. The dendrogram was constructed using UPGMA [27] to discriminate different strains in clusters. For the purpose of evaluating the genetic structure among the populations, the AMOVA analysis of the strains was done using the GenAlEx version 6.2 software. The POPGENE software version 1.32 was also used to calculate the Shannon’s and Nei’s index of diversity. 

## 4. Conclusions

Strains living a common area or near to each other have genetic relationships and genetic exchanges during sexual reproduction, which explains the UPGMA results illustrated by the dendrogram clusters of strains according to their geographical location. The massive genetic diversity of the organisms provides scientists with good opportunities to find new bioactive compounds. Although morphological characteristics are useful for the detection the species and genetic diversity, they depend on environmental conditions and vary under diverse conditions and they are not precise enough to detect the strains and populations, hence the use of the ISSR and RAPD molecular marker have proven to be more powerful methods to distinguish strains geographically. 

## Supplementary Materials

Supplementary File 1

## References

[1] Hughes D.A. (1999). Effects of dietary antioxidants on the immune function of middle-aged adults. Proc. Nutr. Soc..

[2] Grossa G.J., Lockwood S.F. (2004). Cardioprotection and myocardial salvage by a disodium disuccinate astaxanthin derivative (CardaxTM). Life Sci..

[3] Samuel F.L., Marc S.P., Stanley L., Hazen Z.B., Ferenc Z. (2006). The effects of oral Cardax™ (disodium disuccinate astaxanthin) on multiple independent oxidative stress markers in a mouse peritoneal inflammation model: influence on 5-lipoxygenase *in vitro* and *in vivo*. Life Sci..

[4] Kim J., Nam S., Kim B., Choi W., Lee J., Kim W., Choi Y. (2010). Astaxanthin Improves Stem Cell Potency via an Increase in the Proliferation of Neural Progenitor Cells. Int. J. Mol. Sci..

[5] Cianciola E., Popolizio T., Schneider C., Lane C. (2010). Using Molecular-Assisted Alpha Taxonomy to Better Understand Red Algal Biodiversity in Bermuda. Diversity.

[6] Park Y., Lee J.K., Kim N. (2009). Simple Sequence Repeat Polymorphisms (SSRPs) for Evaluation of Molecular Diversity and Germplasm Classification of Minor Crops. Molecules.

[7] Bittencourt-Oliveira M.C., Massola J.N.S., Hernandez-Marine M., Romo S., Moura A.N. (2007). Taxonomic investigation using DNA fingerprinting in *Geitlerinema* species (Oscillatoriales, Cyanobacteria). Phycol. Res..

[8] Arif I., Bakir M., Khan H., Al Farhan A., Al Homaidan A., Bahkali A., Al Sadoon M., Shobrak M. (2010). A Brief Review of Molecular Techniques to Assess Plant Diversity. Int. J. Mol. Sci..

[9] Hall M.M., Vis M.L. (2002). Genetic variation in *Batrachospermum helminthosum* (Batrachospermales, Rhodophyta) among and within stream reaches using intersimple sequence repeat molecular markers. Phycol. Res..

[10] Bornet B., Antoine E., Francoise S., Marcaillou Le., Baut C. (2005). Development of sequence characterized amplified region markers from inter simple sequence repeat fingerprints for the molecular detection of toxic phytoplankton *Alexanndrium catenella* (Dinophyceae) and *pseudo -Nitzschia Pseudodelicatissiam* (Bacillariophyceae) from French coastline waters. J. Phycol..

[11] Dayananda C., Sarada R., Kumar V., Aswathanarayana R.G. (2007). Isolation and characterization of hydrocarbon producing green alga *Botryococcus braunii* from Indian freshwater bodies. Electron. J. Biotechnol..

[12] House D.L., Sherwood R.A., Vis L.M. (2008). Comparison of three organelle markers for phylogeographic inference in *Batrachospermum helminthosum* (Batrachospermales, Rhodophyta) from North America. Phycol. Res..

[13] Zietkiewicz E., Rafalski A., Labuda D. (1994). Genome fingerprinting by simple sequence repeat (SSR) anchored polymerase chain reaction amplification. Genetics.

[14] Bornet B., Branchard M. (2001). Nonanchored Inter Simple Sequence Repeat (ISSR) Markers: Reproducible and Specific Tools for Genome Fingerprinting. Plant Mol. Biol. Rep..

[15] Bardakci F. (2001). Random Amplified Polymorphic DNA (RAPD) Markers. Turk. J. Biol..

[16] Martı´nez R., An˜ı´barro C., Ferna´ndez S. (2006). Genetic variability among *Alexandrium tamarense* and *Alexandrium minutum* strains studied by RAPD banding pattern analysis. Harmful Algae.

[17] Prevost A., Wilkinson M.J. (1999). A new system of comparing PCR primers applied to ISSR fingerprinting of potato cultivars. Theor. Appl. Genet..

[18] Singh R., Zaki N.M., Ting N., Rosli R., Soon-Guan T., Leslie L.E., Ithnin M., Cheah S. (2008). Exploiting an oil palm EST database for the development of gene-derived SSR markers and their exploitation for assessment of genetic diversity. Biologia.

[19] Bhau B.S., Medhi K., Sarkar T., Saikia S.P. (2009). PCR based molecular characterization of *Nepenthes khasiana* Hook Pitcher. Plant Genet. Resour. Crop. Evol..

[20] Ferna´ndez M., Figueiras A., Benito C. (2002). The use of ISSR and RAPD markers for detecting DNA polymorphism, genotype identification and genetic diversity among barley cultivars with known origin. Theor. Appl. Genet..

[21] Zhao K.G., Zhou M.Q., Chen L.Q. (2007). Genetic diversity and discrimination of *Chimonanthus praecox* (L.) Link germplasm using ISSR and RAPD markers. HortScience.

[22] Rahimmalek M., Bahreininejad B., Khorrami M., Tabatabaei B.E.S. (2009). Genetic Variability and Geographic Differentiation in *Thymus daenensis* subsp. *daenensis*, an Endangered Medicinal Plant, as Revealed by Inter Simple Sequence Repeat (ISSR) Markers. Biochem. Genet..

[23] Dellaporta S.L., Wood J., Hicks J.B. (1983). A plant DNA mini-preparation Version II. Plant Mol. Biol. Reptr..

[24] Bornet B., Antoine E., Bardouil M., Marcaillou-Le Baut (2004). ISSR as new markers for genetic characterization and evaluation of relationships among phytoplankton. J. Appl. Phycol..

[25] Rohlf F. (2001). NTSYS-pc Numerical Taxonomy and Multivariate Analysis System.

[26] Huangfu C., Song X., Qiang S. (2009). ISSR variation within and among wild *Brassica juncea* populations: implication for herbicide resistance evolution. Genet. Resour. Crop. Evol..

[27] Sokal R.R., Sneath P.H.A. (1963). Principles of Numerical Taxonomy.

