# Supplementary figures and images for: Studies on the Genetic Variation of the Green Unicellular Alga Haematococcus pluvialis (Chlorophyceae) Obtained from Different Geographical Locations Using ISSR and RAPD Molecular Marker
Source: Molecules. 2011 Mar 22;16(3):2599–608. doi: 10.3390/molecules16032599 (PMC6259651; doi:10.3390/molecules16032599)

**Pooled ISSR and RAPD Data**  
**UPGMA with Jacard's Coefficient**

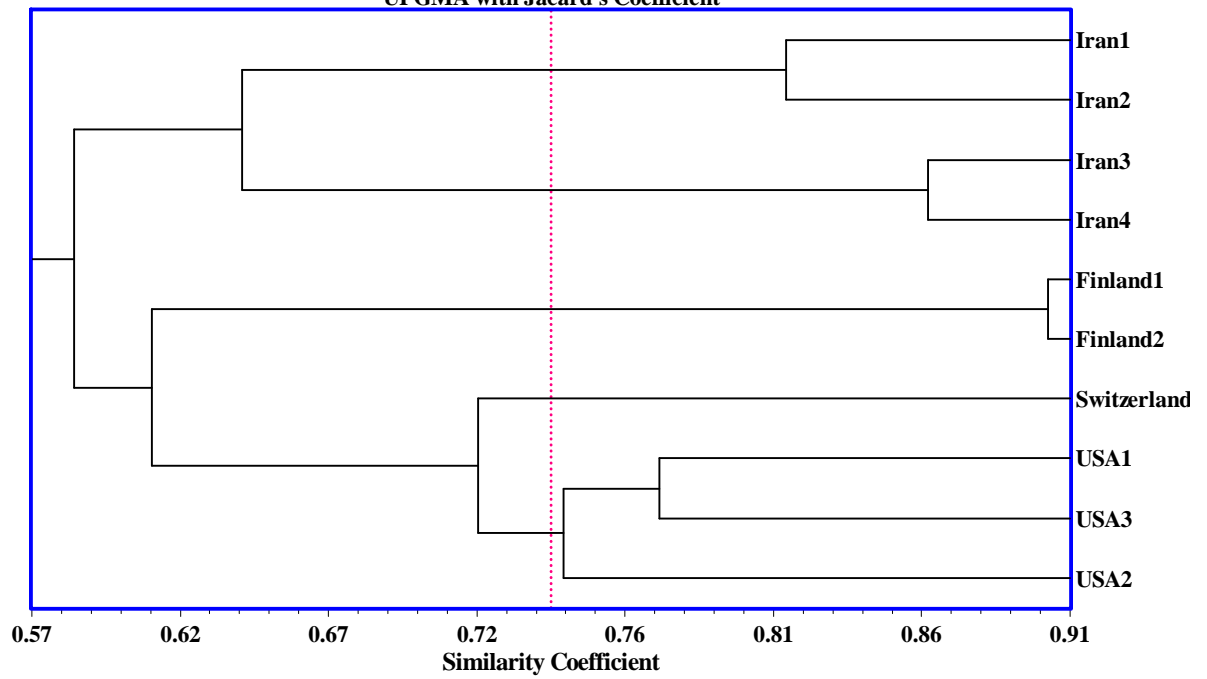

Supplement: Supplementary File 1 [file molecules-16-02599-s001.pdf]
